# Laparoscopic resection of prescral and obturator fossa schwannoma

**DOI:** 10.1590/S1677-5538.IBJU.2015.0091

**Published:** 2017

**Authors:** Marcos Tobias-Machado, Alexandre Kiyoshi Hidaka, Letícia Lumy Kanawa Sato, Igor Nunes Silva, Pablo Aloisio Lima Mattos, Antonio Carlos Lima Pompeo

**Affiliations:** 1 Departamento de Urologia, Faculdade de Medicina do ABC, Santo André, São Paulo, Brasil

## Abstract

**Introduction:**

Pelvic Schwannoma is an extremely rare event. Laparoscopic approach for radical resection on pelvic region already has been described in the literature. However, with better image quality provided by optic in the laparoscopy we can assure an improvement in this kind of approach for tumor resection.

**Objective:**

Our goal is to describe and evaluate the results of one laparoscopic resection of presacral and obturator fossa tumor.

**Materials and Methods:**

We present a case of a 60-year-old man with progressive congestion in the right inferior member and CT scan revealing a mass with miscellaneous content located behind of the right iliac vessels and right obturator nerve. Exploratory transperitoneal laparoscopy was indicated. During laparoscopy it was possible to see the mass between the spermatic cord and external iliac artery. We made the identification and preservation of iliac vessels and obturator nerve. Resection of the tumor was performed carefully, allowing the safe removal of the specimen with complete preservation of the iliac vessels and obturator nerve.

**Results:**

Mean operative time of 150 minutes. No perioperative complications occurred. Two days of hospital stay. Posterior histopathological exam confirmed that the mass was a Schwannoma.

**Conclusion:**

The maximization of the image in the laparoscopic surgery offers dexterity and capacity of dissection required for complex mass dissection on pelvic region.

## ARTICLE INFO

Available at: http://www.intbrazjurol.com.br/video-section/20150091-tobias-machado_et_al/

Int Braz J Urol. 2017; 43 (Video #8): 566-6

